# A Novel Actin mRNA Splice Variant Regulates ACTG1 Expression

**DOI:** 10.1371/journal.pgen.1003743

**Published:** 2013-10-03

**Authors:** Meghan C. Drummond, Karen H. Friderici

**Affiliations:** Department of Microbiology and Molecular Genetics, Michigan State University, East Lansing, Michigan, United States of America; University of North Carolina at Chapel Hill, United States of America

## Abstract

Cytoplasmic actins are abundant, ubiquitous proteins in nucleated cells. However, actin expression is regulated in a tissue- and development-specific manner. We identified a novel cytoplasmic-γ-actin (*Actg1*) transcript that includes a previously unidentified exon (3a). Inclusion of this exon introduces an in-frame termination codon. We hypothesized this alternatively-spliced transcript down-regulates γ-actin production by targeting these transcripts for nonsense-mediated decay (NMD). To address this, we investigated conservation between mammals, tissue-specificity in mice, and developmental regulation using C2C12 cell culture. Exon 3a is 80% similar among mammals and varies in length from 41 nucleotides in humans to 45 in mice. Though the predicted amino acid sequences are not similar between all species, inclusion of exon 3a consistently results in the in the introduction of a premature termination codon within the alternative *Actg1* transcript. Of twelve tissues examined, exon 3a is predominantly expressed in skeletal muscle, cardiac muscle, and diaphragm. Splicing to include exon 3a is concomitant with previously described down-regulation of *Actg1* in differentiating C2C12 cells. Treatment of differentiated C2C12 cells with an inhibitor of NMD results in a 7-fold increase in exon 3a-containing transcripts. Therefore, splicing to generate exon 3a-containing transcripts may be one component of *Actg1* regulation. We propose that this post-transcriptional regulation occurs via NMD, in a process previously described as “regulated unproductive splicing and translation” (RUST).

## Author Summary

Actin is a well-studied protein that plays an essential role in nearly all cell types. Cytoplasmic actins are considered to be ubiquitously expressed in most tissues of the body with the exception of developing skeletal muscle, where muscle specific actins are up-regulated and γ-actin is repressed. Interest in the regulation of this transcript led to the hypothesis that intron retention is responsible for down-regulation of cytoplasmic γ-actin in skeletal muscle during development. Since the publication of the sequence of γ-actin cDNA over two and a half decades ago, no additional splice variants or cDNAs of this gene have been described. In this paper, we identify an alternatively spliced transcript in muscle that allowed us to elucidate how the γ-actin is downregulated during the important transition from myoblast to differentiated muscle cells. This is the first description of regulation of an actin transcript by regulated unproductive splicing and translation.

## Introduction

All mammals express six isoforms of actin: α-cardiac (*Actc1*, NM_009608), α-skeletal (*Acta1*, NM_009606), α-aortic (*Acta2*, NM_007392), γ-enteric (*Actg2*, NM_009610), β-cytoplasmic (*Actb*, NM_007393), and γ-cytoplasmic (*Actg1*, NM_009609). Each actin is encoded on a separate chromosome but the coding sequence of the actins are 71% identical and there is 92% amino acid sequence identity between actin proteins. This degree of conservation is indicative of intolerance of these proteins to changes in amino acid composition, presumably because of the large number of proteins that interact directly with actin. Although the coding sequences are similar between actin isoforms, the genomic architecture of actin isoforms differs between the cytoplasmic (six exons), smooth muscle (nine exons), and cardiac and skeletal isoforms (seven exons). The genomic sequence of *Actg1* was first described in 1986 by Erba and colleagues [1] and no splice variants of this gene have been reported.

In most dividing cells the two cytoplasmic actins are expressed at high levels. For example, mature skeletal and cardiac muscle derive from myoblasts, which express high levels of β- and γ-actin in their undifferentiated form. However, during differentiation, and in mature skeletal and cardiac muscle, the cytoplasmic actins are down-regulated to comprise only a small fraction of the total actin content, and α-skeletal and α-cardiac actins, respectively, become the predominant isoforms [2]–[4]. Nevertheless *Actg1*-null mice demonstrate that γ-actin is crucial for the normal function of mature skeletal muscle, as its complete absence results in a progressive myopathy in adult mice [5], [6].

C2C12 mouse myoblast cell culture is widely used to study the expression and regulation of genes during skeletal muscle development. In this system, myoblasts proliferate until induced to differentiate either via serum-starvation or substitution of horse serum into the growth medium. Differentiation of myoblasts involves exit from the cell cycle, fusion with neighboring cells, elongation into myotubes, and movement of the nuclei to the periphery of the myotubes. Subsequent maturation is characterized by bundling of α-actin thin filaments to form myofibrils. Down-regulation of *Actb* in differentiated C2C12 cells was previously attributed to a 40 nucleotide long conserved element in the 3′ UTR of the *Actb* transcript [7]. In contrast, *Actg1* down-regulation was proposed to involve inhibition of splicing of intron 3 from the primary *Actg1* transcript, thus preventing the production of a mature RNA [2].

A potentially relevant mechanism for post-transcriptional down-regulation is Regulated Unproductive Splicing and Translation [8]. RUST occurs by alternative splicing to include a regulatory exon which either contains, or creates via frameshift, a premature termination codon (PTC). Introduction of a PTC results in subsequent degradation of the mRNA by nonsense-mediated decay (NMD). Recent evidence suggests that as many as 4% of transcripts include alternatively spliced exons with PTCs, though there is some debate as to whether this splicing is functional or an artifact of highly active splicing factors in cells and therefore should be considered transcriptional noise [9], [10]. To help resolve this, Zhang and colleagues proposed criteria for deciding whether a splicing event is functional or noise. Briefly, they have suggested that alternative splicing is likely to be functional if the exon is: 1) conserved among species, 2) developmentally regulated, and 3) tissue specific [11].

We identified an alternatively spliced exon in *Actg1* transcripts from mouse skeletal muscle cDNA. This alternative transcript includes a novel 45 bp exon (exon 3a) located in the middle of *Actg1* intron 3 that introduces a PTC. A similar alternative *Actg1* transcript is found in skeletal muscle from multiple other mammals. In mouse, splicing to include this exon is regulated in a tissue and development specific manner. Here we demonstrate that inclusion of exon 3a results in post-transcriptional down-regulation of *Actg1* by RUST.

## Results

### Identification of a novel *Actg1* transcript

**Figure 1:**
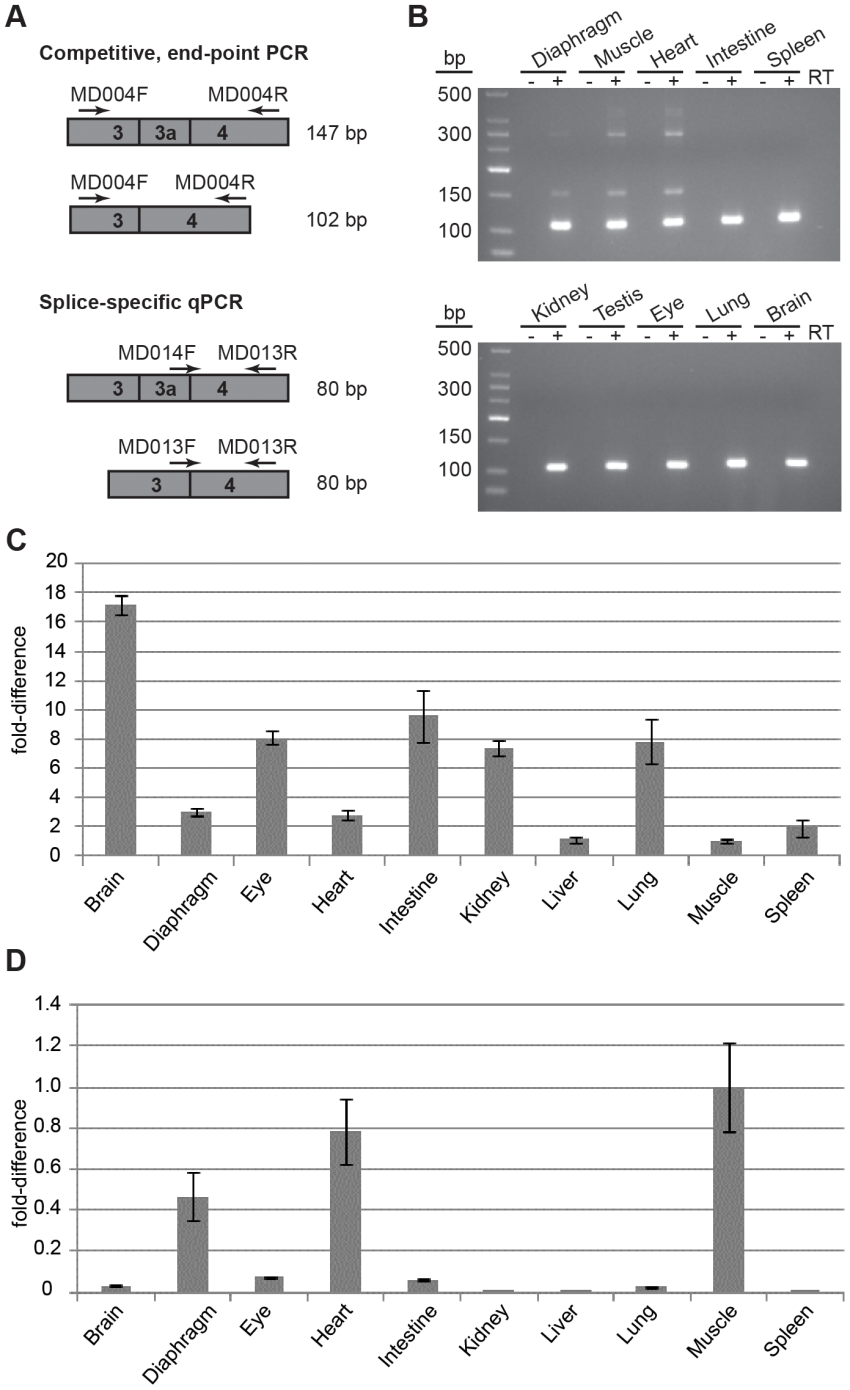
Splicing to include exon 3a into the *Actg1* transcript is tissue specific. (**A**) Two PCR-based assays were employed to screen for the presence of *Actg1* – competitive end-point PCR and splice-specific qPCR. *Actg1*-specific primers were designed to amplify either both the normal and alternative transcripts in a single reaction (competitive end-point PCR), or specifically the normal or alternative transcripts in separate reactions (splice-specific qPCR). (**B**) The competitive end-point PCR assay was used to amplify all *Actg1* transcripts in various tissues from adult mouse. The larger PCR products at ∼390 bp and ∼290 bp are intermediate spliceforms of *Actg1*. The 147 bp product represents alternatively spliced, exon 3a-containing *Actg1* transcripts, while the 102 bp product represents normally spliced *Actg1* transcripts. (**C**) Expression data from qPCR to amplify normal *Actg1* (exon 3 – exon 4) shows a high degree of variability of γ-actin expression between tissues. (**D**) Splicing to include exon 3a, as measured by qPCR is primarily limited to skeletal and cardiac muscle, with very low levels in the brain, eye, and intestine. All qPCR data is normalized to *Rplp0* and *Rrn18s* expression and presented as a fold-difference to skeletal muscle.

**Figure 2:**
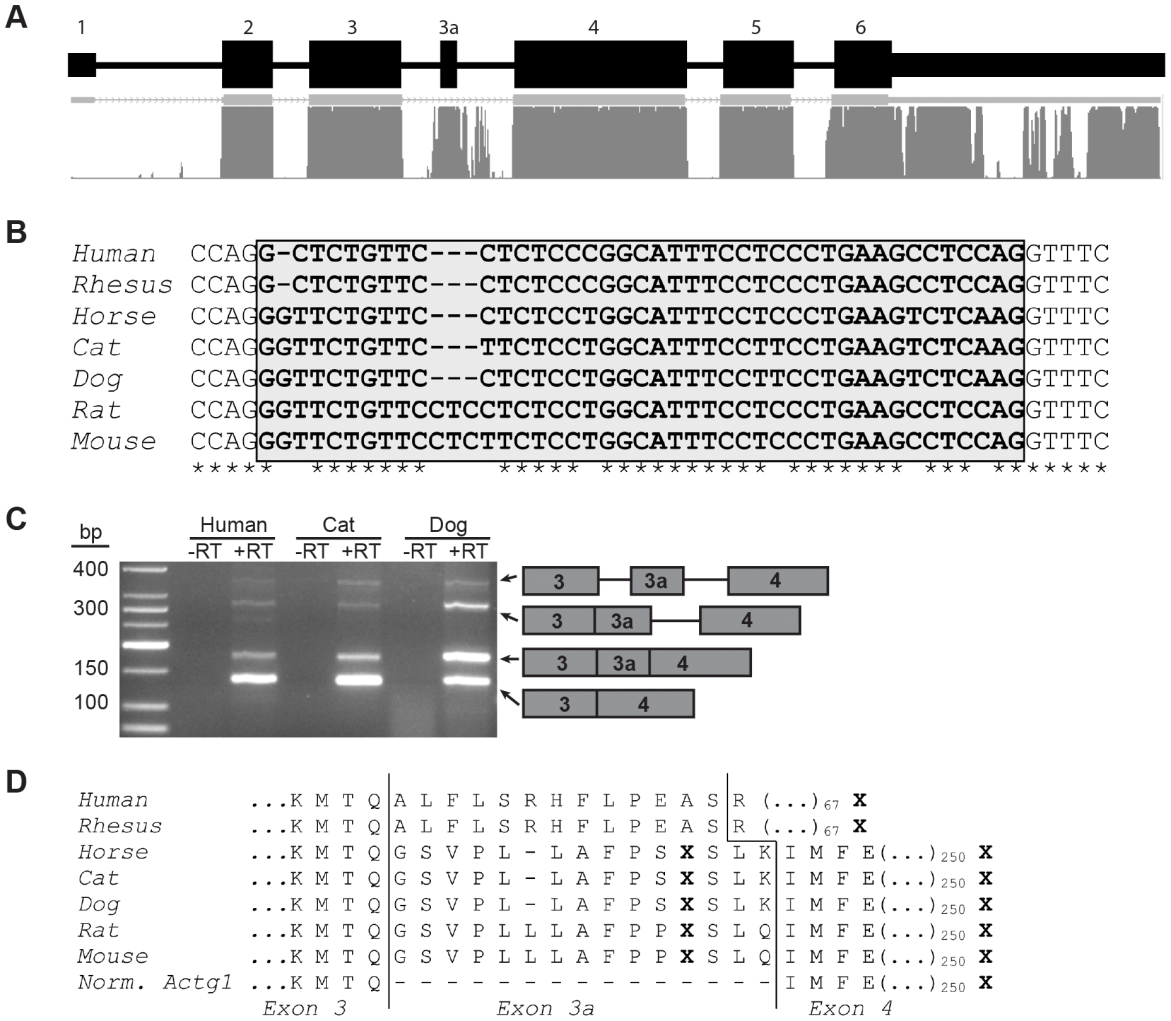
Exon 3a and flanking sequence display a high degree of conservation among mammals. (**A**) *Actg1* gene structure and mammalian conservation based on UCSC Genome Browser. (**B**) Alignment of the nucleotide sequence of exon 3a (shaded box) in seven mammals shows that exon 3a is flanked by canonical splice sites in all species and is 80% conserved. (**C**) Species- and isoform- specific primers were used to amplify across the exon 3 – exon 4 junction of *Actg1* in humans, cat, and dog skeletal muscle cDNA. Similar to the competitive end-point PCR assay described in Figure 1, larger products are present corresponding to intermediate spliceforms. The primary *Actg1* transcript corresponds to a 135 bp product, whereas inclusion of exon 3a results in a larger, 177 bp product (176 bp in human), as visualized on a 3% agarose gel. (**D**) Predicted translated products resulting from inclusion of exon 3a creates an in-frame stop codon (X) and is nearly perfectly conserved in all species with the exception of primates. In primates, an additional 1 bp deletion generates a frameshift resulting in degeneration of amino acid conservation of exon 3a and introduction of a premature stop codon (X) 68 amino acids into exon 4.

While investigating γ-actin expression in a knock-in mouse model harboring a targeted mutation in exon 4 of *Actg1*, we identified a novel, alternatively spliced *Actg1* transcript in wild-type animals. PCR amplification of skeletal muscle cDNA designed to amplify mouse *Actg1* exon 3 to 4 (Figure 1A, Table 1), but to not allow amplification of *Actb* and *Acta1*, yielded an unexpected product. In addition to the predicted 102 bp product, an amplicon of 147 bp was observed in skeletal muscle, heart and diaphragm (Figure 1B). Sequencing of the 147 bp product from skeletal muscle revealed an alternatively spliced transcript that includes a 45 bp exon located in intron 3, which we designate exon 3a (Figure 2A,B). It should be noted that in addition to the PCR products corresponding to the two alternatively spliced transcripts, larger amplicons that were not present in the no-RT controls were observed. Gel extraction and Sanger sequencing of these products revealed these were partially spliced products which included regions of intron 3 upstream and downstream of exon 3a (Figure 2C). In mouse, exon 3a is flanked by canonical splice acceptor and donor sites, and inclusion predicts introduction of an in-frame termination codon (Figure 2B,D). A BLAST search of the NCBI mouse EST database revealed transcripts that included exon 3a (http://blast.ncbi.nlm.nih.gov/Blast.cgi).

**Table 1:** The sequence of primers used in this study.

	Primer	Sequence (5′→3′)	Target
	MD003F	AGGCCAACAGAGAGAAGATGACT	Hs *ACTG1*
	MD003aR	CGTCTCCAGAGTCCATGACA	Hs *ACTG1*
**Competitive**	MD004F	GAACCCCAAAGCTAACAGAGAG	Mm *Actg1*
**End-Point PCR**	MD004R	CAGATGCATACAAGGACAGCAC	Mm *Actg1*
	MD006F	AACCAACGGTGAGAAGATGACT	Cf/Fc *Actg1*
	MD006R	ATCCCCAGAGTCCATGACAATA	Cf/Fc *Actg1*
	MD013F	CAGAGAGAAGATGACGCAGATA	Mm *Actg1* - normal splice only
	MD013R	CATGACAATGCCAGTGGTG	Mm *Actg1*
	MD014F	TCCCTGAAGCCTCCAGATAA	Mm *Actg1* - alternative splice
	2musPpiaF	ACTGTCGCTTTTCGCCG	Mm *Ppia*
**qPCR**	2musPpiaR	GCTGTCTTTGGAACTTTGTCTG	Mm *Ppia*
	MD015F	ACAACAAGCTGCGTGAGGAC	Mm *Rrn18s*
	MD015R	CAGTGGTCTTGGTGTGCTGA	Mm *Rrn18s*
	MD016F	GCGACCTGGAAGTCCAACTA	Mm *Rplp0*
	MD016R	GCTCCCACAATGAAGCATTT	Mm *Rplp0*

Primers were designed using Primer3 to amplify cDNA from either human (Hs), mouse (Mm), or dog (Cf) and cat (Fc).

### Exon 3a-containing transcripts are enriched in muscle

To determine if this transcript was present in other tissues of the adult mouse, cDNA was prepared from diaphragm, skeletal muscle, heart, intestine, spleen, kidney, testis, eye, lung, and brain from adult mice. Only skeletal and cardiac muscle were positive for the presence of alternatively spliced transcripts in addition to the normal *Actg1* transcript, which was the major product amplified (Figure 1B).

To quantify expression of *Actg1* isoforms, we designed primers compatible with qPCR to specifically amplify transcripts resulting from either an exon 3 - exon 4 (normal transcript) or an exon 3a - exon 4 splice (alternative transcript) (Figure 1A). Unlike competitive end-point PCR, the qPCR assay permits detection of low levels of the alternatively spliced *Actg1* 3a transcript. Using this method, we were able to obtain the relative abundance of normal *Actg1* and alternative *Actg1* transcript levels across tissues (Figure 1C,D). We found that brain exhibited the highest level of normal *Actg1*, whereas skeletal muscle and liver had the least. As expected from the results of the end-point PCR, skeletal muscle had the highest level of alternative *Actg1*.

The relative level of the alternative transcript was compared to that of the normal transcript using combined data from three qPCR experiments. The efficiencies of the PCR reactions for the normal and alternative transcript were within <1% of each other and the threshold was set at 0.2 for both reactions. This analysis revealed average **Δ**Ct values of 20.8 for the normal and 22.7 for the alternative transcripts in skeletal muscle corresponding to a 3.5∶1 ratio of normal to alternatively spliced *Actg1* (Table 2).

**Table 2:** Ct values for a skeletal muscle cDNA control demonstrate no plate-to-plate variability.

Experiment	Threshold	Avg Ct for Normal Splice	Avg Ct for Alternative Splice
**Tissue Panel**	0.2	20.75	22.62
**C2C12 Timecourse**	0.2	21.01	22.70
**C2C12 CHX Treatment**	0.2	20.95	22.65
**Combined Average**	–	20.90	22.70

For each qPCR plate, a skeletal muscle cDNA control sample was included to assess plate-to-plate variability for every primer pair. For the *Tissue Panel* experiment Ct values represent three skeletal muscle cDNA biological replicates in addition to the control skeletal muscle cDNA sample. Alternatively spliced transcripts constitute approximately 30% of steady-state *Actg1* mRNA in adult skeletal muscle. Two technical replicates were averaged for every biological replicate in qPCR experiments.

### Exon 3a is highly conserved among mammals

Evolutionary conservation of nucleotide sequence is typically indicative of functional significance. While no conservation of intron 3 is detected in fish or chicken cytoplasmic actin, the nucleotide sequence of *Actg1* intron 3 is highly conserved among mammals. Specifically, the region containing the 45 bp alternatively spliced exon and flanking splice sites are 80% identical between humans and mice (Figure 2A,B). To determine if splicing of the *Actg1* transcript is an evolutionarily conserved event *in vivo*, we prepared cDNA from human, dog, and cat skeletal muscle total RNA. Species- and isoform-specific primers were designed similar to the competitive end-point PCR assay for mouse described above and outlined in Figure 1A. Splicing to include exon 3a was observed in skeletal muscle cDNA from the species assayed (Figure 2C). All PCR products were sequenced to confirm the imputed exon 3a sequence (Figure 2B, human, cat, dog). Alignment of sequences obtained from the UCSC genome browser (http://genome.ucsc.edu) indicates that inclusion of exon 3a is predicted to introduce an in-frame PTC in non-primate mammals. In humans and rhesus, exon 3a is 41 nt in length and results in a frameshift of the *ACTG1* coding sequence, thus creating a PTC in exon 4. While the amino acid sequence of the predicted polypeptide generated by inclusion of exon 3a is highly conserved in non-primate mammals, the frameshift generated by the 4 nt deletion in primates results in a complete loss of this conservation (Figure 2D).

### Developmental regulation of *Actg1* alternative splicing

**Figure 3:**
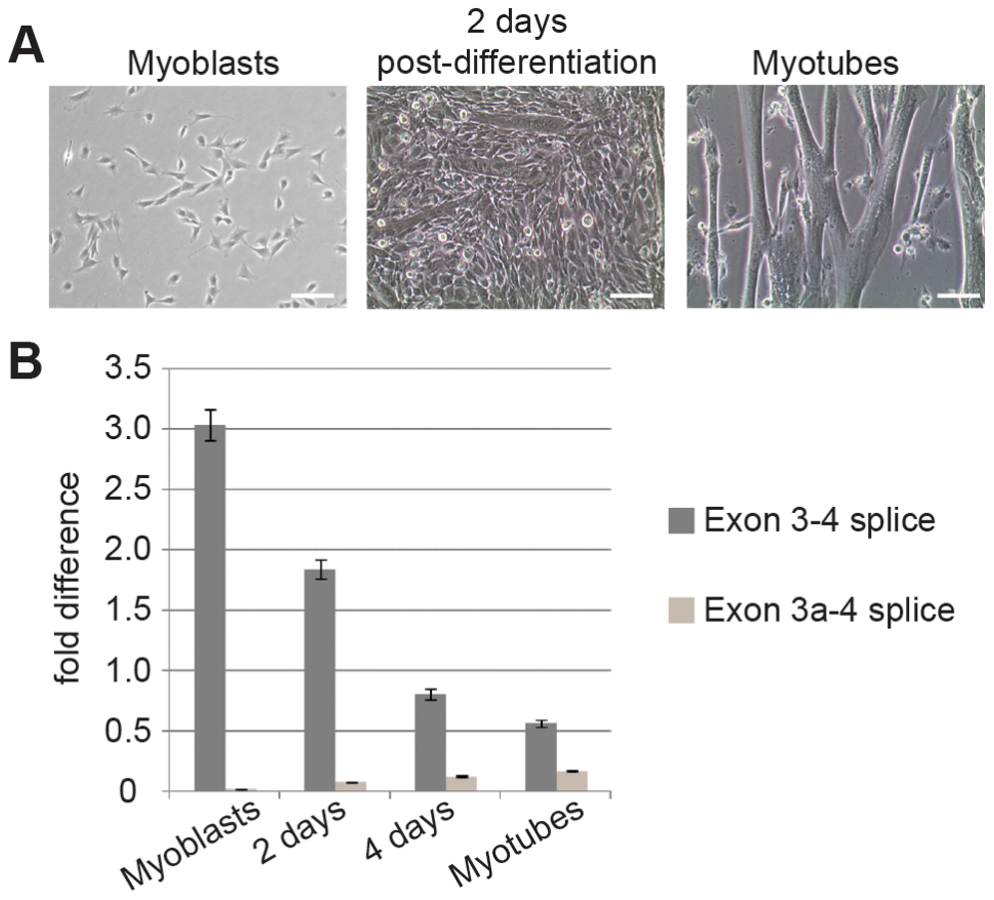
Splicing to include exon 3a is a developmentally regulated event in skeletal muscle. (**A**) Microscopy of cell cultures before, during and after differentiation. C2C12 myoblasts were grown to 70% confluence and induced to differentiate in DMEM+10% horse serum. Partially differentiated cultures containing both myoblasts and myotubes were observed by 2 days post-differentiation. After 48 hours, medium was replaced with DMEM+2% horse serum and 10 µM Ara-C and cultured for an additional 4 days. (**B**) qPCR of RNA harvested in Trizol showed that concurrent with a decrease in normal *Actg1*, splicing to generate alternative *Actg1* increases during differentiation into myotubes. Expression of both the normal and alternative transcripts was normalized to *Ppia* and is presented as fold-difference compared to skeletal muscle. A two-tailed type 2 Student's T-test was used to compare expression differences between time points. For all time points compared, p<0.0001.

To investigate the potential function of the alternative *Actg1* transcript in a relevant tissue, we utilized the well-characterized C2C12 mouse myoblast cell line as a proxy for skeletal muscle development. C2C12 cells are frequently used to study transcriptional and proteome changes during the differentiation of myoblasts into myotubes (shown in Figure 3A) [12], [13]. Using these cells, we first asked if *Actg1* alternative splicing is developmentally regulated. Total RNA was isolated from myoblasts prior to addition of differentiation medium and at 2 day intervals after addition of differentiation media. qPCR was used to determine expression levels of normal and alternatively spliced *Actg1*. In agreement with previous studies by Lloyd and Gunning [2], our results demonstrate that normal *Actg1* is down-regulated during myoblast differentiation (Figure 3B). These data also reveal that concurrent with the decrease in normal *Actg1* expression, alternatively spliced *Actg1* transcripts increase during differentiation (Figure 3B).

### Exon 3a-containing transcripts are exported to the cytoplasm, but no corresponding protein product is present

**Figure 4:**
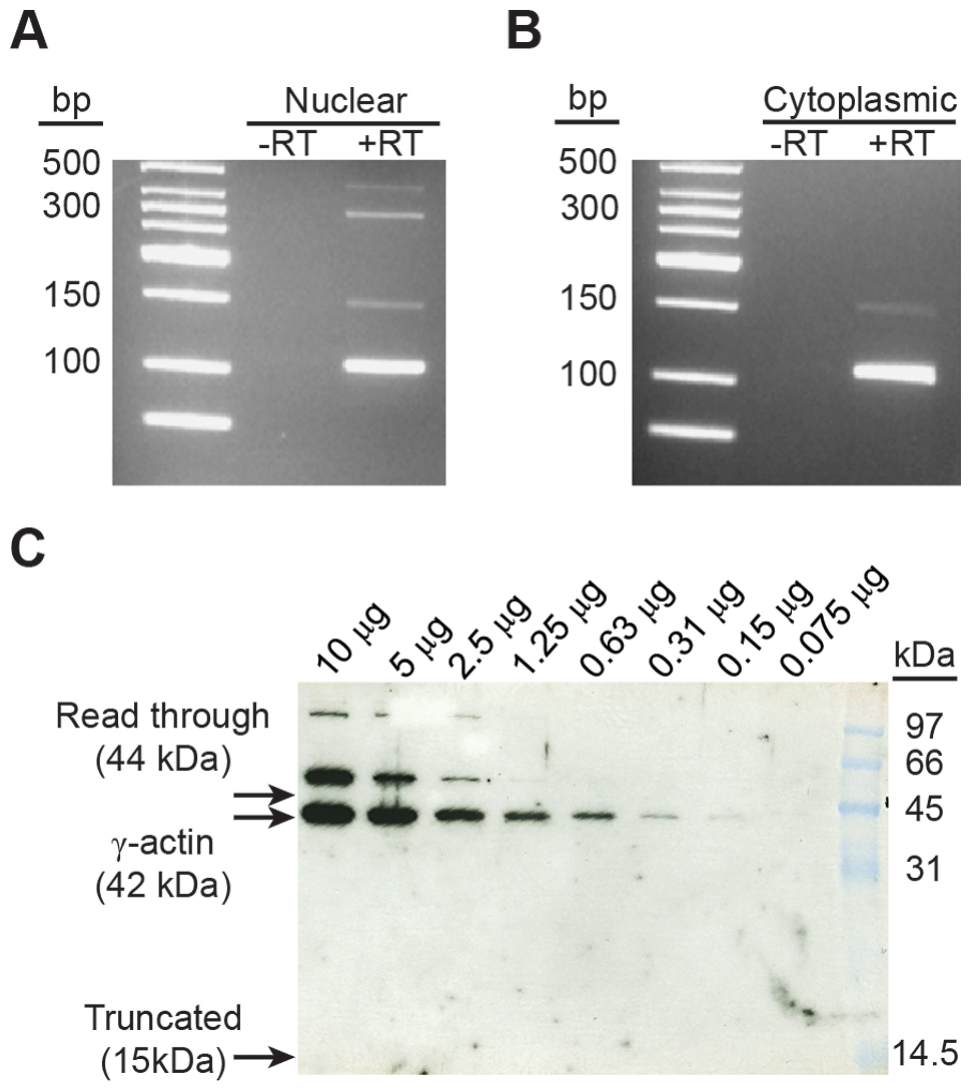
Exon 3a containing alternatively spliced *Actg1* is exported to the cytoplasm, but does not produce a stable protein. Nuclear (**A**) and cytoplasmic (**B**) RNA fractions were harvested from C2C12 myotubes and evaluated for the presence of alternative *Actg1* using competitive, end-point PCR. All *Actg1* spliceforms, including partially spliced transcripts, were present in the nuclear fraction, however, only the normal and exon 3a transcripts were observed in the cytoplasmic fraction. (**C**) Western blot using an anti-γ-actin specific antibody was used to probe mouse skeletal muscle lysate for the presence of a protein product corresponding to the inclusion of exon 3a. Usage of the in-frame stop codon in exon 3a would generate a 15 kDa protein. Alternatively, read-through of the stop codon would increase the size of the ACTG1 protein by 2 kDa, resulting in a 44 kDa protein. Neither of these protein products is present. A γ-actin protein at 52 kDa is observed and likely represents a post-translational modification. Molecular weight marker is prestained Low Range (BioRad).

Given the lack of conservation in the amino acid sequence generated by inclusion of exon 3a (Figure 2D), we hypothesized that a protein product is not produced from the alternative *Actg1* transcript. To address this, we first sought to determine if the alternative transcript is exported to the cytoplasm and therefore available for translation. Total RNA was isolated from both cytoplasmic and nuclear fractions of mature myotube cultures. Using competitive end-point PCR, we found that the nuclear fraction contained all splice products including putative splicing intermediates, whereas only the normal and alternatively spliced *Actg1* transcripts were present in the cytoplasmic fraction (Figure 4A,B).

Knowing that the alternative transcript is exported to the cytoplasm, we used western blotting to detect a protein product corresponding to either the use of the premature stop codon or a read-through of the stop codon. Because the levels of alternative transcript in the skeletal muscle samples exceed that of the myotubes in culture, we used skeletal muscle lysate for this experiment. Using an anti-γ-actin specific antibody directed against the N-terminus of the polypeptide, we probed for the presence of a protein product from alternatively spliced *Actg1* transcripts. Based on the qPCR data, there should be a 3∶1 ratio of normal ACTG1 to alternative ACTG1 (Table 2). We loaded 1∶2 serial dilutions to determine our lower-limit of detection by western blot, beginning with 10 µg of total protein. No band corresponding either to usage of the termination codon (15 kDa) or a read-through of the termination codon in exon 3a (45 kDa) was detected (Figure 4C). We did observe a larger, 52 kDa protein in these samples which is consistent in size with previously described modifications of cytoplasmic and skeletal muscle actins, specifically mono-sumoylation [14] or mono-ubiquitination [15].

### Inhibition of nonsense-mediated decay results in an increase of exon 3a-containing transcripts

**Figure 5:**
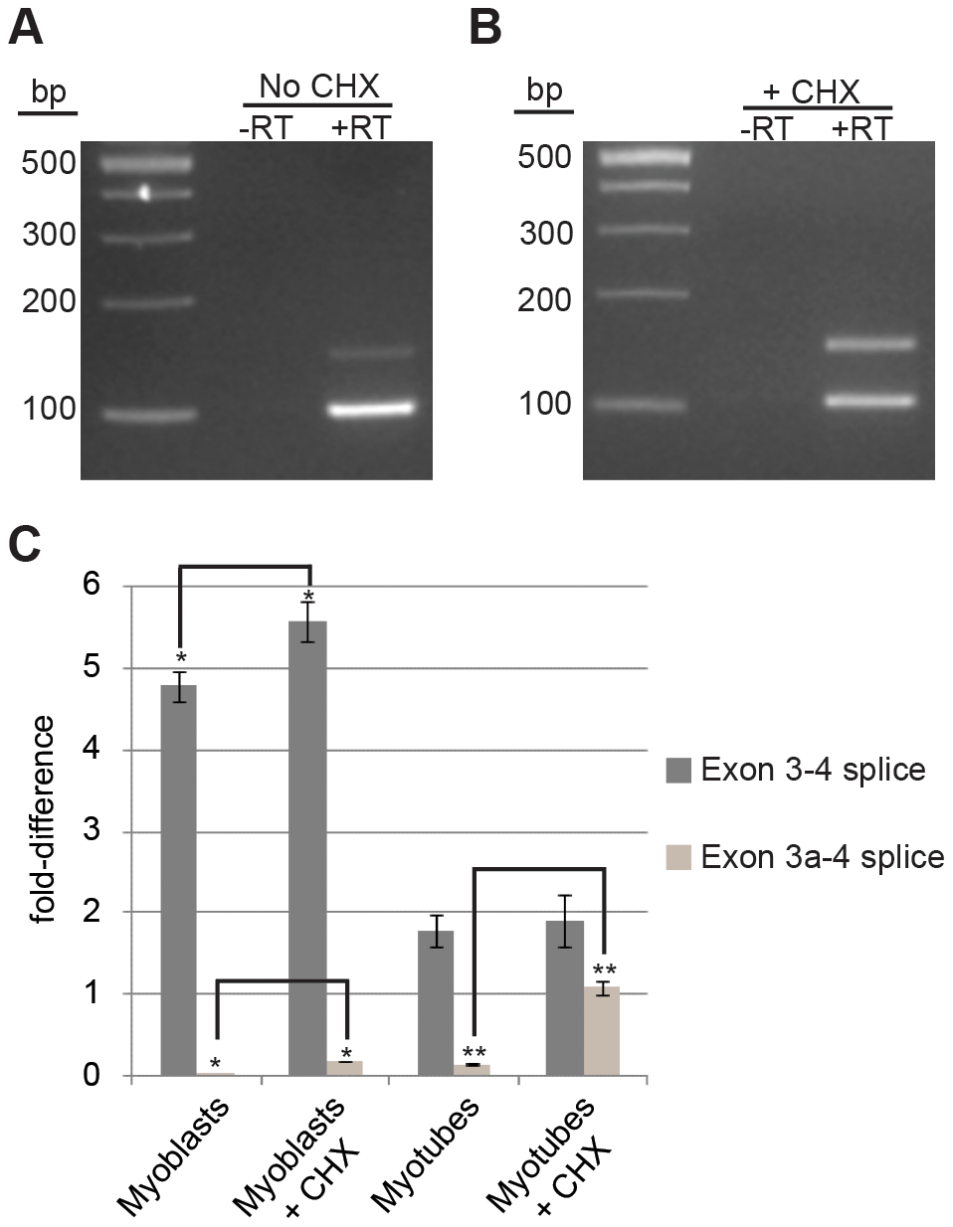
Cycloheximide (CHX) treatment of myotubes results in a 7-fold increase in alternative *Actg1*. (**A,B**) Competitive end-point PCR and (**C**) qPCR were used to evaluate relative abundance in untreated (**A,C**) and CHX treated cells (**B,C**). Expression of both the normal and alternative transcripts were normalized to *Ppia* and are presented as fold-difference compared to skeletal muscle. A two-tailed type 2 Student's T-test was used to compare expression differences between samples. * p<0.05, ** p<0.001.

We reasoned that exon 3a is alternatively spliced to post-transcriptionally down-regulate expression of *Actg1*. To address the hypothesis that exon 3a represses translation of *Actg1* by targeting the transcript for NMD, we treated cells with cycloheximide to block translation. Cycloheximide targets the small ribosomal subunit and can be used to inhibit translation-dependent NMD of PTC-containing transcripts [16]. Cultures of proliferating myoblasts and mature myotubes were treated with either 40 µg/mL cycloheximide in ethanol or an ethanol-only control for three hours in otherwise standard growth conditions. A three-hour treatment with cycloheximide resulted in an approximately 7-fold increase of exon 3a transcripts as measured by qPCR (Figure 5). These data strongly suggest that exon 3a targets the transcript for translation-dependent NMD. Furthermore, they indicate that splicing to include exon 3a is a frequent event in mature myotubes, given the rapid increase in the relative abundance of the alternatively spliced product.

## Discussion

In this study, we identified a novel *Actg1* splice variant enriched in cardiac and skeletal muscle. We propose that production of this alternative transcript is regulated and functional. Despite the fact that cytoplasmic actins are well-studied and widely used as reference genes, this is the first report of alternative splicing for any actin transcript. Why might exon 3a containing transcripts have been overlooked? The most divergent sequence for distinguishing between actin transcripts reside in the 5′ and 3′ UTRs. PCR primers designed to specifically amplify one isoform are typically in the UTRs and inclusion of a small exon, such as the 41–45 nt exon 3a, is likely to be overlooked in a large, >1 kb PCR product. Furthermore, short PCR products in which a 41–45 nt difference could be resolved are unlikely to involve exons 3–5 of the actin transcript because the high degree of nucleotide similarity between actin isoforms in these exons. Given that the alternative exon appears to be specific to γ-actin, a product corresponding to the alternative transcript would be lost among the abundance of the other actin cDNAs amplified.

Previous studies suggested that the phenomenon of retention of intron 3 in the primary *Actg1* RNA would be responsible for the down-regulation of γ-actin during differentiation of myoblasts [2], [17]. Here we add support for this hypothesis and provide evidence for regulated splicing to incorporate an additional exon situated in the middle of intron 3 which is sufficient for down-regulation of *Actg1* via a mechanism not previously shown for an actin transcript. Cycloheximide inhibition of translation results in an elevation in the level of exon 3a-containing transcripts, a finding that is consistent with translation-dependent NMD. Cycloheximide is commonly used as an inhibitor of NMD [16], [18], [19], however there exists the possibility that the increase in exon 3a-containing transcripts is the result of another effect of the cycloheximide treatment rather than a direct correlation with inhibition of the NMD pathway. Therefore, assuming no additional effects of treatment with cycloheximide, we hypothesize that γ-actin is down-regulated via alternative splicing to introduce a PTC, leading to the degradation of *Actg1* transcripts via NMD [8], [20].

We considered the possibility that the presence of the alternatively spliced *Actg1* transcript is the result of ‘noisy splicing’. Large-scale analyses indicate that the majority of alternatively spliced transcripts are likely generated in error because of their low abundance across multiple tissues and lack of correlation with expression differences in the genes examined [9], [21], [22]. However, several examples of RUST as a mechanism of post-translational down-regulation exist [23]–[26]. Using the guidelines established by Zhang and colleagues to distinguish between noisy and functional splicing, we evaluated whether inclusion of exon 3a in *Actg1* transcripts is likely to be spurious [11], [22]. A primary feature of ‘noisy splicing’ is lack of conservation of the alternative splice form in other species. However, we find that, similar to the normal coding exons of *Actg1*, exon 3a is highly conserved in mammals. Furthermore, in agreement with criteria for functional splice variants, we demonstrated that the alternative splice form is abundant only in skeletal and cardiac muscle, and that this splicing event is differentially regulated in developing myotubes.

Splicing to include exon 3a in *Actg1* transcripts maintains the proper reading frame in most species, however in primates exon 3a is only 41 nucleotides, thereby creating a frameshift. This subtle difference in the sequence of exon 3a between primates and lower mammals further supports the regulatory hypothesis in that it is the generation of a PTC and not a translated product that is evolutionarily conserved. Previous studies of RUST indicate that this type of regulation is not only conserved across species, but is typically found as a regulatory mechanism for members of the same gene family. We believe RUST may post-transcriptionally regulate both cytoplasmic actins, γ and β. Both cytoplasmic actins have a similar genomic structure and similar to γ -actin, β-actin has high degree of conservation in a portion of intron 3 [27]. In addition, it was recently demonstrated that an expression construct utilizing the β-actin promoter in combination with the 3′ UTR resulted in high level expression in skeletal muscle [28], [29]. It is possible the conserved region of intron 3 of β-actin may confer an additional level of regulation in addition to the previously reported conserved 40 nt element in the 3′UTR [7]. The genomic structure of the non-cytoplasmic actins is markedly different from the cytoplasmic actins, and lack the high degree conservation observed in Actg1 and Actb intron 3. Therefore, RUST is an unlikely mechanism of regulation for the non-cytoplasmic actin isoforms.

The premise of RUST seems counter-intuitive as a regulatory mechanism, since the most efficient means of down-regulation would be at the transcriptional level. However, Soergel *et al* note that the production of large transcripts in any instance can be an inherently wasteful endeavor, as introns can constitute up to 95% of a primary RNA transcript [30], [31]. It is possible that RUST can serve as a mechanism to quickly modulate expression of genes that are typically highly expressed, form very stable transcripts, or are essential in the cell. When a particular environmental or physiologic change dictates that only moderate levels of protein are required, production can be down-regulated without entirely switching-off transcription. In such an instance, post-transcriptional degradation of a portion of excess transcripts produced via NMD may be more energy efficient for the cell than either switching on or off transcription or post-translational degradation of unnecessary proteins. This model is suitable for γ-actin in muscle, as it is expressed at high levels in proliferating myoblasts, but is also necessary at lower levels in differentiated myotubes and developed skeletal muscle. RUST due to inclusion of exon 3a is unlikely to be the only mechanism of regulation for cytoplasmic actin transcripts. Though oftentimes used as a constitutive promoter in *in vitro* systems, the *Actb* and *Actg1* promoters may also supply further control to the down-regulation of actin at a transcriptional level. Furthermore, retention of the entire intron 3 of the immature *Actg1* message may contribute to delayed processing of mature transcripts [2]. Indeed, the increased abundance of incompletely spliced heterogeneous RNA observed when exon 3a-containing transcripts were present (figures 1B, 2C, 4a) may suggest a role for intron retention in down-regulation.

While the alternative transcript is enriched in skeletal and cardiac muscle, we were also able to detect very low levels in brain, eye, and intestine; tissues which also expressed normal *Actg1* at high levels. Regulation of β-actin synthesis was previously shown to be influenced by the levels of actin monomers in cells treated with an actin depolymerizing agent, latrunculin A [32]. We speculate that γ-actin expression may also involve an auto-regulatory mechanism by which an excess of free actin monomers in the cell induces splicing to include exon 3a of the *Actg1* transcript, thus temporarily halting production in cell types which normally require high levels of actin. In such a situation, very low levels of exon 3a-containing transcipts would be expected in tissues with a high cytoplasmic actin content.

**Figure 6:**
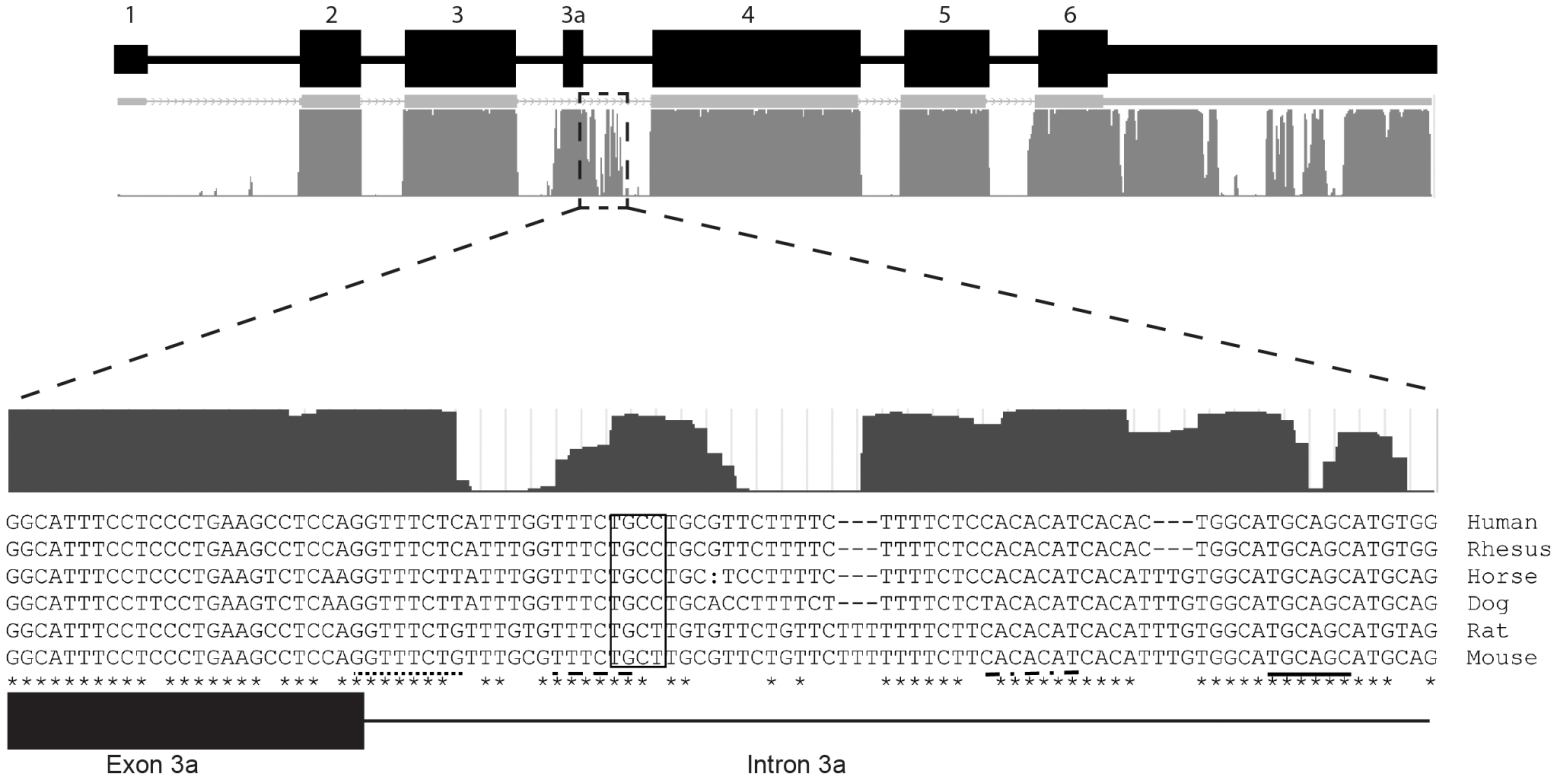
The intronic sequence immediately 3′ of exon 3a is highly conserved. The UCSC mammalian conservation track shows a high degree of conservation in the 3′ flanking sequence. ESE Finder v3.0 was used to identify consensus sequences for multiple splicing enhancer elements in human and mouse, including SRSF1 (dot-dash line), SRSF2 (dotted line), SRSF5 (dashed line), SRSF6 (solid line). All predicted ESEs shown in this figure are considered “high-score motifs” because the threshold is greater than the baseline threshold for each motif: SRSF1 = 3.658 (minimum 1.956); SRSF2 = 4.893 (minimum 2.383); SRSF5 = 3.877 (minimum 2.670); SRSF6 = 4.713 (minimum 2.676). In addition, a muscleblind-like 1 YCGY motif (box) is found 3′ of the exon 3a [34].

In skeletal and cardiac muscle, exon 3a splicing may be regulated by splicing enhancer or splicing repressor elements. The intronic region immediately adjacent to the 5′ donor site of exon 3a is well conserved among mammals (Figure 6). During differentiation of myoblasts, multiple transcription and splicing factors are required to coordinate changes in gene expression crucial for differentiation. As such, the intronic conservation immediately 3′ of exon 3a may contain recognition motifs for splicing enhancers or repressors. Indeed, an exon splicing enhancer prediction software, ESE Finder v3.0, identified several splicing enhancer recognition motifs (Figure 6). Of particular interest are the recognition motifs for SF2/ASF and SC35, which were shown previously to effect alternative splicing of β-tropomyosin in a tissue-specific manner [33].

In closing, this report documents the first identification and characterization of an alternatively spliced actin transcript. These data provide evidence for the dynamic regulation of *Actg1* and further functional evidence for RUST.

## Materials and Methods

### Bioinformatics

Sequence ascertainment and analysis was performed using UCSC Genome Browser/BLAT (http://www.genome.ucsc.edu/), Ensembl! (http://www.ensembl.org), Sequencher 6.0 (Gene Codes Corp.), and Clustal X2 (http://www.clustal.org). Primers were designed using Primer3 software (http://frodo.wi.mit.edu/). Potential exon splicing enhancers were identified using ESEFinder v3.0 (http://rulai.cshl.edu/cgi-bin/tools/ESE3/esefinder.cgi)

### Animals and tissue preparation

All animals were maintained according to Michigan State University IACUC and NIH guidelines. Tissue samples were harvested from three 1 year old C57Bl/6J mice, snap frozen on dry ice, and stored at −80°C. Prior to use for RNA or protein isolation, samples were chopped into 100–200 mg pieces. Cat and dog skeletal muscle samples were provided courtesy of Dr. John Fyfe (Michigan State University). Human skeletal muscle total RNA was purchased from Ambion (catalog AM7982; Austin, TX).

### Cell culture

All cell culture media and supplements were purchased from Invitrogen (Carlsbad, CA), unless noted otherwise. C2C12 myoblast cells were purchased from ATCC (Manassas, VA; CRL-1772) and propagated in DMEM containing 10% heat-inactivated fetal bovine serum and 2 mM L-glutamine. Differentiation of myoblasts into myotubes was achieved by culturing cells >70% confluent in DMEM supplemented with 10% horse serum in place of fetal bovine serum. Forty-eight hours post differentiation, DMEM with 2% horse serum and 10 µM Ara-C (Sigma, St. Louis, MO) was used to maintain differentiated myotubes and inhibit the proliferation of myoblasts. Cells were maintained at 37°C in 5% CO_2_.

### RNA isolation and cDNA synthesis

Total RNA isolation from tissue and cell culture samples was achieved using TRIzol (Invitrogen, Carlsbad, CA) purification followed by a DNaseI treatment using RNeasy mini-columns (Qiagen, Hilden, Germany). Total RNA was quantified using a NanoDrop (Thermo, Wilmington, DE). cDNA was synthesized using SuperScriptIII reverse transcriptase (Invitrogen, Carlsbad, CA) according to manufacturer's instructions. Reaction volume was 20 µl; for qPCR, 300 ng of RNA was used per reaction, and for endpoint PCR, 1 µg of RNA was used. Following incubation at 55°C for 1 hour, samples were heat inactivated at 75°C for 20 minutes then stored at −20°C until used.

### Nuclear and cytoplasmic RNA isolation

Myotube cultures were lysed rapidly with 1% Triton X-100 in PBS, cell debris and nuclei were gently scraped from the culture dishes and evaluated by brightfield microscopy for the presence of intact nuclei. Samples were centrifuged at 1,000×g for 5 minutes to pellet nuclei. The cytosol-containing supernatant was examined under the microscope to assure the absence of nuclei in the cytosolic fraction prior to the addition of TRIzol LS to the supernatant and TRIzol to the pellet (Invitrogen, Carlsbad, CA). RNA purification of both the cytosolic and nuclear fractions was done according to manufacturer's instructions.

### Competitive end-point PCR amplification

*Actg1* cDNA was amplified using Promega GoTaq polymerase per manufacturer's instructions (Madison, WI) with 5 µM each of species- and isoform-specific primers located in exons 3 and 4 of *Actg1* (Table 1). For most reactions, 28 cycles were sufficient to amplify within the perceived linear range. Products were evaluated on 3% agarose gels containing 0.3 µg/mL ethidium bromide and visualized using BioRad GelDoc System (Hercules, CA). Pixel intensity of the PCR products was quantitated using GelDoc software (BioRad, Hercules, CA) for semi-quantitative analysis.

### qPCR

Quantitative PCR (qPCR) was performed on an ABI7000, using Power SYBR Green (Invitrogen, Carlsbad, CA) as the reporter dye, and data were collected using StepOne Plus software (Applied BioSystems, Carlsbad, CA). Data were analyzed using qbase^PLUS^ software (Biogazelle, Zwijnaarde, Belgium). *Actg1* transcripts were normalized to *Ppia* for experiments using C2C12 cells and to *Rplp0* and *Rrn18s* for experiments using mouse tissues. See Table 1 for primer sequences. Averaged data represent two technical replicates for each of three biological replicates. A two-tailed type 2 Student's T-test was used to compare expression differences between samples.

### Sequencing

Sequence data for exon 3a were obtained by Sanger sequencing on an ABI Prism 3700 DNA Analyzer at the Research Technology Support Facility (Michigan State University) using competitive, end-point PCR (Table 1).

### Cycloheximide treatment

Cycloheximide (Sigma, St. Louis, MO) was dissolved in 100% ethanol at a stock concentration of 40 mg/mL and added to growth medium at a final concentration of 40 µg/mL. Cells were incubated in cycloheximide containing medium for 3 hours and immediately harvested in TRIzol for RNA isolation and cDNA synthesis as described above. Each experiment was repeated three times.

### Protein isolation

Approximately 100 mg of skeletal muscle from a 1 year old mouse was lysed using a Polytron rotor homogenizer in lysis buffer containing 100 mM KCl, 10 mM PIPES, 5 mM EGTA, 1% Triton X-100 and Complete Protease Inhibitors (Roche, Basel, Switzerland), and incubated on ice for 1 hour to allow further lysis. Protein content in the total lysate was determined using a Bradford assay (BioRad, Hercules, CA).

### Western blotting

Proteins were separated via SDS-PAGE on discontinuous 12% bis-acrylamide gels [10]. Proteins were transferred in 10 mM Tris pH 7.4, 100 mM glycine, 15% methanol (transfer buffer) at 4°C overnight at a constant current of 5 mAmp onto polyvinylidene difluoride (PVDF) membranes (BioRad, Hercules, CA). Membranes were incubated in PBS (pH 7.4) containing 5% non-fat milk and 0.025% Tween-20 (blocking buffer) for one hour at room temperature. A previously validated rabbit polyclonal anti-γ-actin specific antiserum raised against the first 15 amino acids of the polypeptide [5] was diluted 1∶10,000 in blocking buffer. Membranes were incubated with primary antiserum for 2 hours at room temperature. Goat polyclonal anti-rabbit IgG-HRP conjugated secondary antibody (Sigma, St. Louis, MO) was used at 1∶3,000 in blocking buffer for one hour at room temperature. Proteins were detected using an ECL Detection Kit (GE Healthcare, Waukesha, WI) with Amersham Hyperfilm MP autoradiography film (GE healthcare, Waukesha, WI).
